# Mapping 340B Funds Flow Through Contract Pharmacies

**DOI:** 10.1177/00469580251404363

**Published:** 2025-12-23

**Authors:** Anthony M. DiGiorgio, Parisa Jahangirizadeh, Deborah Williams

**Affiliations:** 1 The University of California, San Francisco, CA, USA; 2 Zuckerberg San Francisco General Hospital and Trauma Center, San Francisco, CA, USA; 3 The University of California, Berkeley, CA, USA; 4 Health Policy Insights, LLC

**Keywords:** 340B, Health Policy, contract pharmacy, covered entities, pharmacy benefit managers, third-party administrators, drug pricing

## Abstract

The 340B Drug Pricing Program, established in 1992, was designed to help safety-net healthcare providers stretch scarce resources by allowing them to purchase outpatient medications at significant discounts. However, the program’s dramatic expansion, particularly following the Affordable Care Act, and the rise of contract pharmacy arrangements have led to increasing scrutiny regarding its current function and impact. This study examines the flow of funds within the modern 340B ecosystem, with a particular focus on contract pharmacies, pharmacy benefit managers (PBMs), third-party administrators (TPAs), and vertically integrated health systems. Using a detailed mapping of financial transactions, the analysis identifies key areas where profit incentives diverge from the program’s original intent. Covered entities (CEs) often generate substantial profits by purchasing drugs at 340B-discounted rates and receiving standard reimbursements from public and private payers, including Medicare and commercial insurers. These profits are not required to be reinvested in care for underserved populations. Additionally, the use of contract pharmacies and TPAs has introduced significant complexity, increasing the risks of drug diversion and duplicate discounts, particularly with Medicaid and Medicare rebate programs. Vertically integrated PBM pharmacy chains dominate the landscape, often capturing outsized revenue. Legal and regulatory ambiguities further complicate oversight and enforcement. This paper provides an overview of the current 340B funds flow through contract pharmacies, highlighting areas of misalignment with the program’s mission. In doing so, it informs ongoing legislative and administrative reform efforts, emphasizing the need to re-center the program around its intended beneficiaries, low-income and vulnerable patients.

## Introduction

The rapid growth of the 340B Program has brought intense scrutiny. Its evolving structure, compared to its original outline in 1992, has raised questions about whether it still serves its founding mission. HRSA describes the 340B program as a way for safety-net hospitals and clinics to “stretch scarce federal resources as far as possible, reaching more eligible patients and providing more comprehensive services.”^1^ However, evidence suggests that its expansion has prompted changes that have made the program lucrative for participating hospitals which may influence care patterns. A 2015 GAO analysis found that average Medicare Part B drug spending per beneficiary at 340B hospitals was about $144, more than twice the $60 spent at non-340B hospitals,^2^ indicating a financial incentive to prescribe more or higher-priced drugs. Other studies report large increases in drug claims in specialty areas such as oncology and ophthalmology^3^ and a rise in treatment intensity when physicians join 340B hospitals.^4^ Conversely, some analyses find little difference in generic prescribing rates between 340B and non-340B prescribers.^5^

340B lowers acquisition costs for covered entities (CE) but Medicare and many private insurers reimburse them at standard rates leading to covered entities generating substantial profit margins (the difference between their low acquisition prices and the reimbursement rates paid by government, private payers, or, often, patients). The program does not require these profits to be used for care of low-income patients, and many newer participants provide relatively little uncompensated care.^3^ This combination of high markups and limited transparency has prompted concerns that the program may unintentionally incentivize hospitals to focus on commercially insured patients, where margins are largest, rather than those with Medicaid or no insurance.^6^

The original legislation was meant to help institutions “stretch scarce resources.” This is, of course, not limited to pharmaceuticals. There is only scant evidence of institutions using these resources to improve access.^7^ The program has gradually expanded through both rulemaking and legislative action. Hospitals and their child sites are now the majority of 340B CEs, and newer data shows that these hospitals tend to use the funds to boost financial investments^8^ instead of increasing access to care. A central driver of this transition is the expansion of contract pharmacies, which shifted 340B utilization from hospital-based, physician-administered settings toward retail-pharmacy dispensing of oral drugs. It introduced a pharmacy benefit manager mediated payment flow that adds intermediaries, increases administrative burden, and heightens the risk of misallocated discounts.^7^

During its original drafting, Congress did not want participation by 340B entities that had, in its words, “a minor contract to provide indigent care which represents an insignificant portion of [the entity’s] operating revenues.”^9^ However, given the program’s incentives, it has benefited many institutions where indigent care is, indeed, an insignificant portion of the entity’s operating revenue.^10^ This is largely because the program has been intentionally expanded multiple times to include institutions which do not primarily serve disadvantaged patients, notably large hospital systems. Today, the beneficiaries of the program’s profits are unclear as reflected by numerous court challenges and legislative reform proposals. This study maps the flow of funds through CEs and contract pharmacies within the current 340B program to identify where it diverges from its original objectives and to inform administrative and legislative reforms that can better align it with the public’s needs. While CEs can purchase and administer pharmaceuticals through other pathways, such as physician administered drugs, we focus on contract pharmacy arrangements for this piece.

## 340B Program Overview

Originally passed in 1992, the 340B program allows certain healthcare organizations, including Disproportionate Share Hospitals (DSH), Federally Qualified Health Centers (FQHCs), Ryan White HIV/AIDS grantees, and other federal clinics, to become covered entities (CEs) eligible to purchase outpatient drugs at substantial discounts. While hospitals often dominate the discussion, non-hospital entities are critical to the program’s reach: federally supported clinics made up 28% of all covered entities.^11^ The Affordable Care Act further expanded CE eligibility to additional hospital categories, including free-standing cancer hospitals, critical access hospitals, and other types of rural hospitals, increasing the number of CE sites from around 8000 in 2000 to over 50 000 (when including hospital child sites) by 2020. Today, over 40% of U.S. hospitals are now CEs.

Initially, hospitals could only distribute their medications at an on-site pharmacy. However, HRSA rulemaking expanded this to one off-site contract pharmacy in 1996, and further rulemaking by the Office of Pharmacy Affairs allowed for unlimited off-site contract pharmacies in 2010.^12^ These changes increased the number of off-site pharmacies from 1000 in 2009 to over 25 000 in 2022, contributing to total 340B drug purchases exceeding $60 billion as of 2024.

The recent Minnesota 340B report^13^ highlights many inefficiencies within the program. For example, it disproportionately benefits large, financially secure hospital conglomerates, while some safety-net federal grantees have shown negative revenue on 340B purchases. This calls into question whether the program is truly serving low-income patients. The report also shows specific revenues per fill, such as $400 for insulin and over $3000 for Humira.

Because of the revenues generated by 340B, some hospitals undertake strategic behavior to meet only the minimum 11.75%^14^ DSH-adjusted rate to qualify. This qualification can be maintained for an entire system by meeting the inpatient DSH minimum at a single qualifying inpatient hospital annually. They often expand into more affluent neighborhoods^15^ by buying or building satellite sites, then generate significant revenue by selling 340B drugs to privately insured and Medicare patients. These outpatient satellite hospitals don’t affect the DSH qualifying metrics. Conversely, these hospitals may avoid expansion into low-income neighborhoods. This pattern has led to increased consolidation and higher drug prices, particularly in oncology.^7^ Similar behavior occurs with pharmacies^16^ and Pharmacy Benefit Managers (PBMs). Many PBMs have vertically integrated with contract pharmacies, allowing them to capture some of the difference between the 340B purchase price and the reimbursement received. This type of consolidation has resulted in 75% of 340B CE–pharmacy relationships belonging to just 5 integrated pharmacy–PBM chains.^17^ The 340B arrangement has become unique among drug discount and rebate programs, as the discount now occurs (originally it was a charge back) at the time of purchase rather than later in the supply chain. By contrast, other rebates, such as the Medicaid Drug Rebate Program, are paid out after the point of sale.

With multiple rebates, discounts, and agents in the overall system, it is not surprising that duplicate discounts still occur despite being prohibited. “Duplicate discounts” arise when the same drug receives both the 340B discount and a Medicaid rebate, which federal law expressly prohibits.^18^ Medicaid programs and covered entities must coordinate, often using mechanisms like the Medicaid Exclusion File, to avoid submitting rebate claims for drugs already purchased under 340B pricing. The Inflation Reduction Act (IRA) introduces a further layer of complexity. Under the IRA, Medicare may negotiate Maximum Fair Prices (MFPs) for selected drugs, creating risk of overlap with 340B discounts. To address this, HRSA’s Rebate Model Pilot Program targets duplicate discounts related to 340B pricing, specifically between 340B prices and IRA MFPs and among multiple covered entities.^19^

## Funds Flow

**Figure 1.**
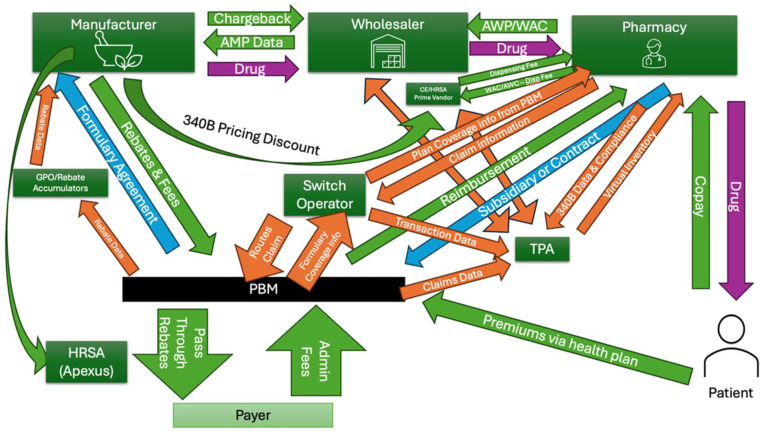
Funds Flow in the Pharmaceutical Supply Chain and 340B Program. Outlines relationships in the pharmaceutical supply chain, highlighting the interactions between manufacturers, wholesalers, pharmacies, pharmacy benefit managers (PBMs), payers, covered entities (CEs), third-party administrators (TPAs), HRSA, and rebate accumulators. The arrow from manufacturer to HRSA (Apexus) indicates manufacturer paid prime vendor fees. HRSA (Apexus) denotes the 340B prime vendor program and its funding via contractor fees. The CE/HRSA prime vendor denotes the distribution/chargeback channel used by CEs to acquire 340B inventory at the negotiated price. Arrows represent the movement of financial transactions (green), data exchange (orange), and drug distribution (purple) across different stakeholders. Baseline PBM/pharmacy admin occurs with or without 340B; the incremental 340B external ops layer (CE → contract-pharmacy + TPA) was ≈$16 per $100 of gross 340B revenue in Minnesota 2023.

See Table 1 for a description of the terms used in pharmaceutical sales and see Figure 1 for a graphical representation of this funds flow. The 340B ceiling price is determined using a formula tied to the Medicaid rebate mechanism. It equals the Average Manufacturer Price (AMP) minus the Unit Rebate Amount (URA), the same components used in calculating Medicaid rebates.^20^ If the calculation (AMP − URA) results in a price of zero, or lower, the program sets a minimum of $0.01 per unit, known as “penny pricing.”^21^ This ensures the price remains operational in the healthcare marketplace and avoids logistical issues. Covered entities, or their agents, purchase 340B drugs from the manufacturer at the discounted price. Apexus, in its role as HRSA Prime Vendor for the 340B program, negotiates the drug discount. Apexus is tasked with helping covered entities obtain sub-ceiling prices and offering tools, education, and technical assistance to promote compliance. Apexus negotiates additional discounts beyond the statutory ceiling and serves as a key resource for both covered entities and other stakeholders seeking guidance on 340B program requirements.

**Table 1.** Timeline of Drug Flow and Financial Transactions in the 340B Program for example Drug X. Outlines the stepwise process involved in the manufacturing, pricing, distribution, dispensing, and financial reconciliation of a drug participating in the 340B Drug Pricing Program.

Timeline	
• Drug Manufacturing	• Dispensing and Payment
• Manufacturer produces Drug X with a list price of $1000	• Pharmacy dispenses Drug X to patient
• Average Manufacturer Price (AMP) is set at $800	• Patient pays copay (eg, $50)
• Wholesale Acquisition Cost (WAC) is set at $850	• PBM reimburses pharmacy $800 (based on negotiated rate)
• Wholesaler Acquisition	• Replenishment and Reconciliation
• Wholesaler purchases Drug X from manufacturer at WAC ($850)	• TPA identifies dispensed drug as 340B eligible
• Wholesaler sets Average Wholesale Price (AWP) at $1050	• TPA initiates replenishment order at 340B price ($600)
• 340B Program Enrollment	• CE billed for replenishment order
• Covered Entity (CE) enrolls in 340B program for Drug X	• Financial Reconciliation
• 340B price calculated at $600 (based on statutory formula)	• Pharmacy retains dispensing fee (eg, $160)
• Virtual Inventory Setup	• CE receives remaining funds ($640)
• Third-Party Administrator (TPA) sets up virtual inventory system	• Manufacturer processes chargeback to wholesaler for 340B discount
• TPA establishes connection with CE, wholesaler, and contract pharmacy	• Rebate Processing
• Virtual Order Placement	• Manufacturer pays rebates to PBM based on utilization data
• TPA places virtual order for Drug X on behalf of CE	• PBM passes through portion of rebates to payer
• Wholesaler ships physical inventory to contract pharmacy	• Audit and Compliance
• Patient Prescription	• TPA conducts routine audit to ensure 340B program compliance
• Patient receives prescription for Drug X from CE’s healthcare provider	• CE reviews financial data and patient benefit
• Prescription sent to contract pharmacy	
• Claim Processing	
• Patient presents prescription at pharmacy	
• Pharmacy submits claim through switch operator	
• PBM processes claim and determines patient’s copay	

When the drug is then sold at contract pharmacies, the pharmacy is paid a dispensing fee. Inventory is maintained by third party administrators (TPAs) which keep track of 340B purchased drugs in a virtual inventory while the contract pharmacy provides the TPA with compliance data.

HRSA requires contract pharmacies to have a system in place to verify that a 340B-acquired drug is dispensed only to eligible patients.^22^ However, there is no centralized method for verifying patient eligibility, which is supposed to be performed at the time the prescription is written. Multiple CEs can claim the same patient, and 1 CE can use multiple TPAs. Dispensing a 340B drug to a non-340B-eligible patient is known as “diversion,” and reliable data on the frequency of this occurrence is lacking.

After the drug is sold, the pharmacy reconciles data from the CE with its own prescription information. Insurance adjudication occurs through a switch operator, which receives claim details from the pharmacy and provides PBM coverage and reimbursement information. The claim has the patient’s insurance coverage information and the PBM has formulary coverage information. The pharmacy then uses TPA software to attribute the prescription to a virtual inventory category.

The switch operator also interfaces with the PBM to route the claim and provides transaction data back to the TPA to update the virtual inventory. The PBM, in turn, provides claim data to the TPA. Because TPA algorithms vary widely in how they track inventory, there have been documented instances of 340B drugs being diverted or duplicated to non340B patients.

PBMs collect fees from payers and premiums from beneficiaries, then reimburse the pharmacy at a pre-negotiated rate. Patients generally pay a copay based on the sale price of the drug rather than the discounted 340B acquisition price.^23^ Meanwhile, the PBM provides rebate data to organizations called “rebate accumulators,” which aggregate and relay data to manufacturers in an effort to avoid duplicate rebates. As noted, PBMs may also supply claims data and coverage details to TPAs and switch operators.

In vertically integrated PBM–pharmacy chains, pharmacies typically pay around AWP minus 20% for the drug, with the remainder of the revenue flowing to the CE. Independent pharmacies, however, are often reimbursed at or near their actual acquisition cost (plus a dispensing fee).

When Medicaid is the payer, reimbursement varies between fee-for-service (FFS) and managed care organizations (MCOs). Under FFS, reimbursement is limited to the 340B ceiling price or actual acquisition cost plus a nominal dispensing fee. Medicaid MCOs, in contrast, often follow commercial insurance practices, creating a possibility of duplicate discounts. Because of the prohibition on double-dipping into Medicaid Drug Rebates (MDRP), some states have “carved out” 340B from their Medicaid MCO benefits, thereby reclaiming MDRP savings while eliminating CEs of potential 340B revenue on Medicaid patients.^24^ Most states have not adopted this carveout, leaving open the risk of duplicate discounts.

Given the large number of intermediaries, each is responsible for its own separate system and incentive. The contract algorithms are not transparent, and many may incentivize higher drug costs. This complexity complicates auditing, and many states do not require pharmacies to classify drug purchases as 340B or non-340B (in fact, some prohibit it),^25^ making the prevention of duplicate discounts exceedingly difficult.

Beyond illustrating complexity, Figure 1 shows how the growth of the 340B program has veered from its intent of helping safety net hospitals, enriching a series of intermediaries instead. In Minnesota’s statewide accounting, covered entities received roughly $750M in 340B revenues, but paid $120M (16%) of that to contract pharmacies and TPAs. Meanwhile, safety-net grantees generated little, and sometimes even negative, margins on 340B.^13^ While baseline retail administrative costs exist in both 340B and non-340B claims, 340B adds in the contract pharmacy, covered entity and TPA layers. These findings empirically link the additional administrative layer in Figure 1 to the lack of benefit relative to the program’s original intent.

## Legal Challenges

Recent court cases^9,26-29^ have brought this tangled web of interacting parties to the forefront. In 2011, *Santa Clara* confirmed that private parties do not have the right to sue manufacturers to enforce 340B claims.^9^ A ruling in a challenge to the Health Resources and Services Administration’s interpretation of the orphan drug exclusion established the limited nature of the agency’s rulemaking authority.^26^ That decision has opened the door to both CEs and manufacturers pursuing various “self-help” strategies that have, in turn, spawned significant litigation. Several manufacturers have, for instance, recently attempted to shift 340B pricing “downstream” by providing it as a rebate, rather than the point of initial sale as an upfront discount. HRSA has deemed this practice illegal and threatened to exclude such manufacturers from Medicare altogether—leading to multiple lawsuits that remain undecided as of this writing.

Additional court cases have attempted to limit the use of contract pharmacies. However, recent rulings in a series of federal courts, including courts located in Arkansas and Louisiana, have reinforced the requirement that drug manufacturers must provide 340B-priced drugs to contract pharmacies, where mandated by state law. These cases are also making their way through the courts, with 1 federal court in West Virginia having concluded that a state statute was preempted under federal law.^27-29^

## Conclusion

The lack of clarity and the numerous legal disputes surrounding 340B underscore the need for reform. Lawmakers recognize this, and several bills have been proposed to address the program’s shortcomings. While the future of 340B remains a hotly debated topic, the funds flow map outlined here illustrates the program’s complexity and the multitude of for profit entities involved. As policymakers consider reforms, they must keep the patient—the intended beneficiary of discounted medications—at the center of any changes.
